# Supplementary material for: Asymmetric migration decreases stability but increases resilience in a heterogeneous metapopulation
Source: Nat Commun. 2018 Jul 30;9:2969. doi: 10.1038/s41467-018-05424-w (PMC6065393; doi:10.1038/s41467-018-05424-w)
Supplement: Supplementary file 1 — Supplementary Information [file 41467_2018_5424_MOESM1_ESM.pdf]

Supplementary Information

Asymmetric migration decreases stability but increases resilience in a heterogeneous metapopulation

Limdi et al.

Supplementary Figures

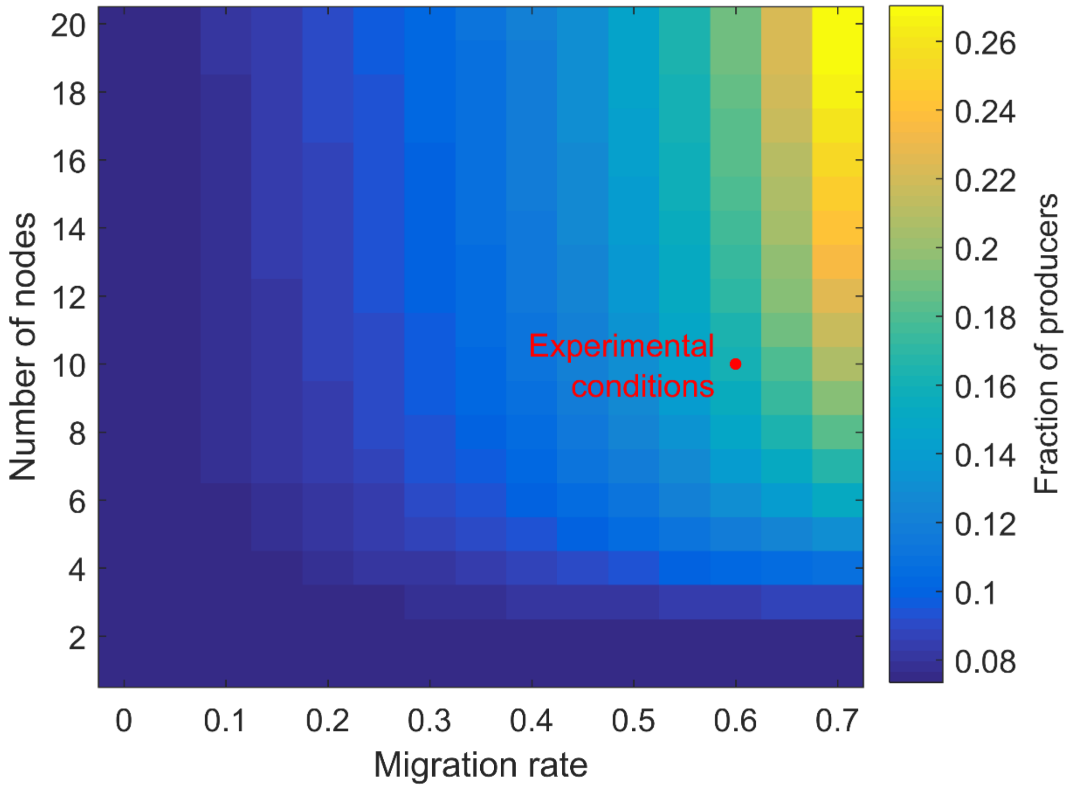

Supplementary Figure 1: For star networks, the overall fraction of producers increases with the number of nodes and migration rate. Theoretically predicted fraction of producers in steady state in the whole metapopulation, for star networks with different number of nodes and migration rates. All other model parameters as in Figure 2 in main text. The red dot shows the parameters for the experimental conditions and the simulations shown in Figure 2.

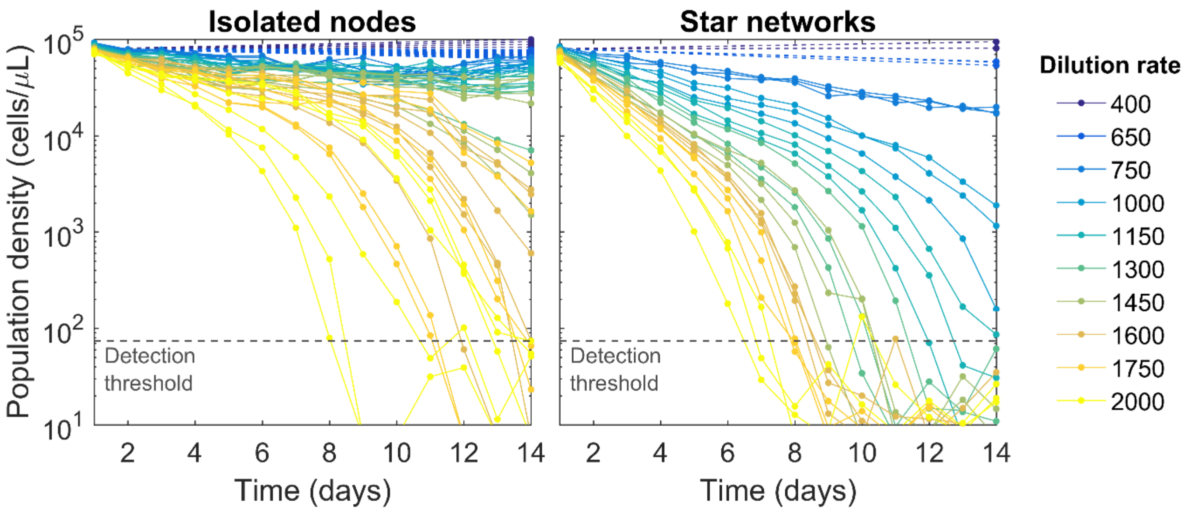

Supplementary Figure 2. Time series show that star networks collapse at lower dilution rates than isolated nodes. Left: Population density over time for isolated nodes at different dilution rates. Dashed lines indicate cases in which only initial and final densities were measured. Data at 14 days correspond to the points shown in main text **Figure 3B**. **Right:** Same as (Left), but for star network (population density is averaged across each network).

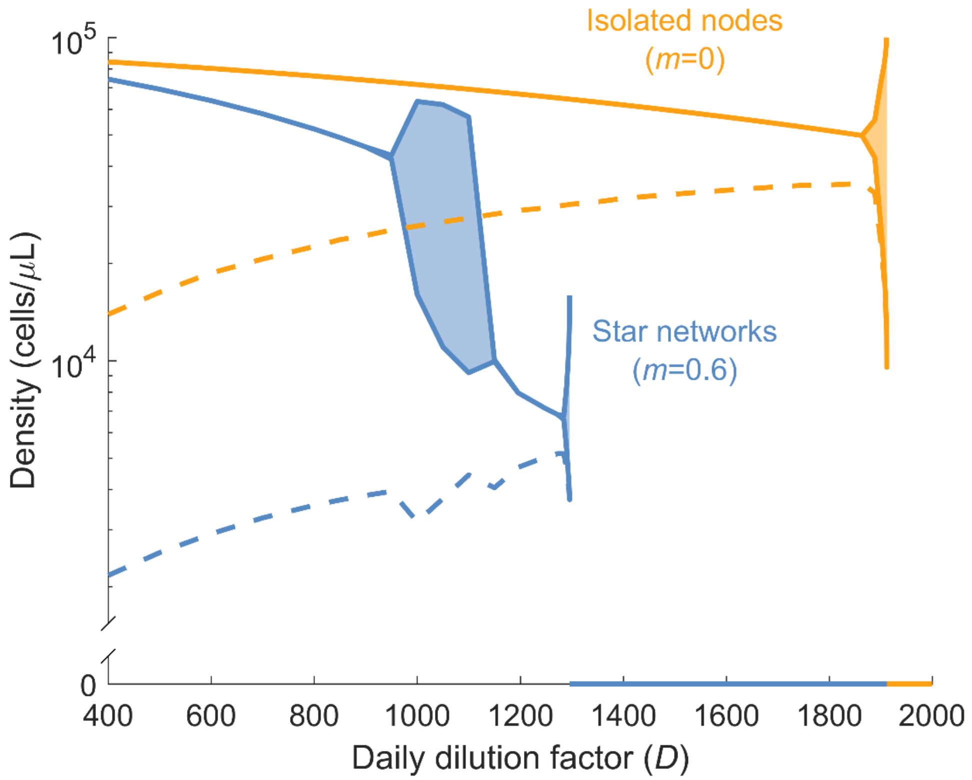

Supplementary Figure 3. Bifurcation diagram for the metapopulation, accounting for oscillatory states.

Solid lines show the average population density in equilibrium as a function of dilution rate (yellow: isolated nodes; blue: 10-node star network). For some dilution factors the system reaches a limit cycle, presenting stable oscillations. These cases are represented by a shaded patch, the top line corresponding to the peak population density and the bottom line corresponding to the valley population density. Dashed lines show the minimum density from which the metapopulation can recover (assuming same producer frequencies and same relative densities between center and side nodes as the population in equilibrium; when oscillations are present, we took the population at the lowest point of the cycle as a reference). Model parameters are as in Fig. 2.

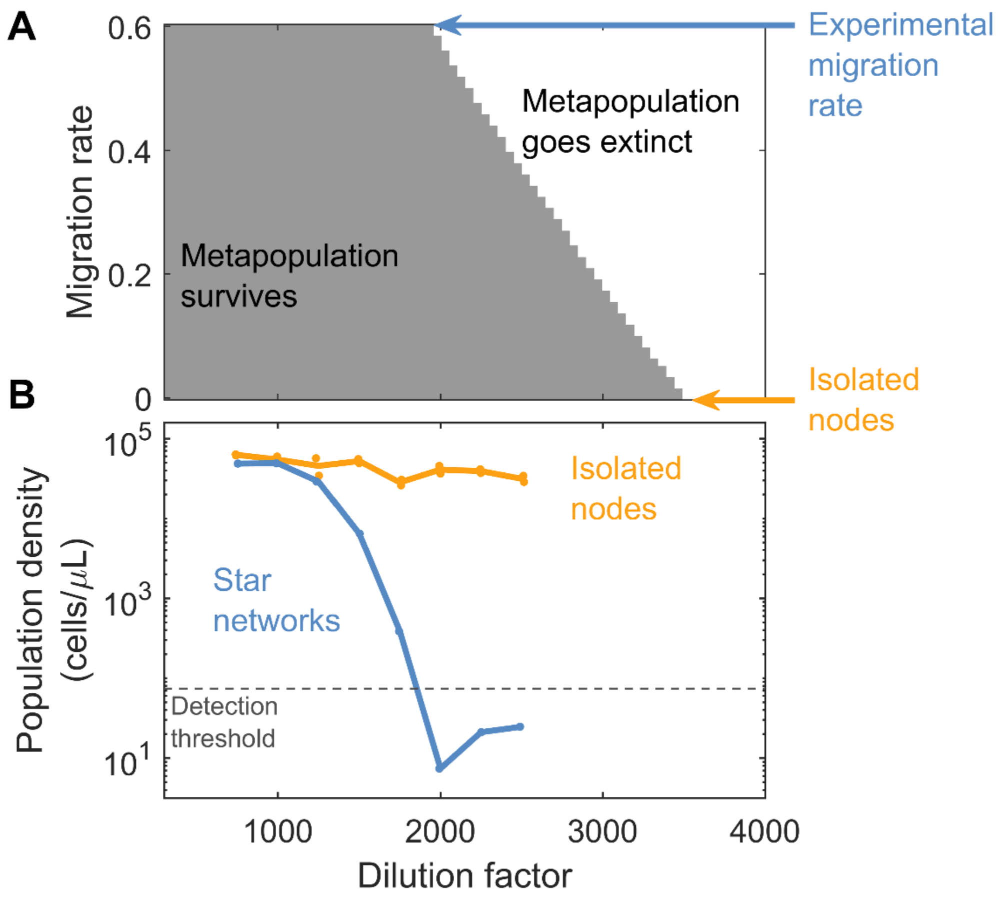

Supplementary Figure 4: Heterogeneous metapopulations are less able to survive challenging environments than isolated populations, also when composed only of producers. (A) Same as Figure 3A in main text (including model parameters), but with 100% producers. **(B)** Same as Figure 3B in main text, but for an experiment with 100% producer cells.

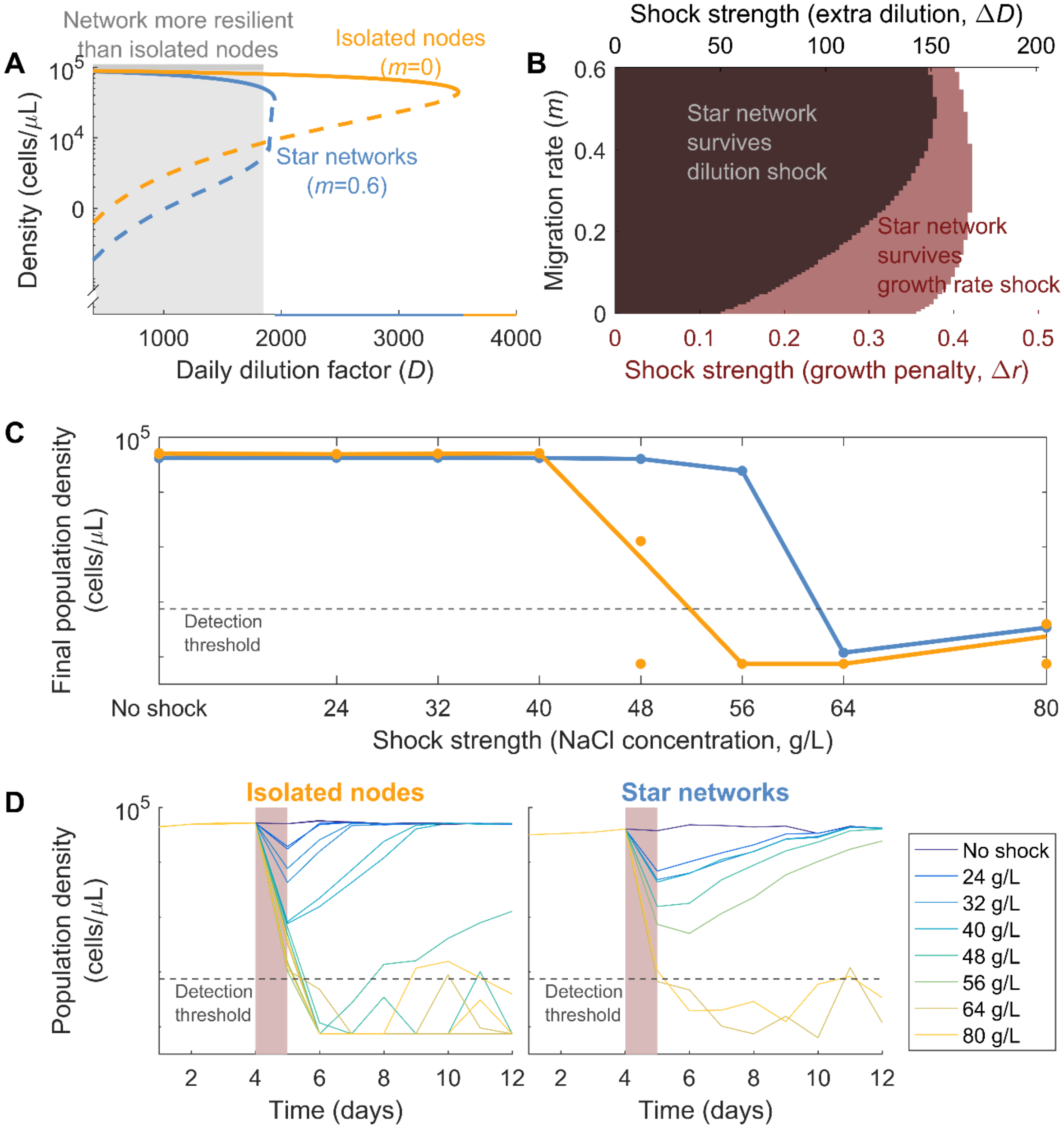

Supplementary Figure 5: Heterogeneous metapopulations are more resilient also when only producers are present. (A) Same as Figure 4A in main text (including model parameters), but with 100% producers. **(B)** Same as Figure 4B in main text, but with 100% producers. **(C)** Experimental data showing the total population density after 12 days vs strength of the osmotic shock, for star metapopulations (blue) or isolated nodes (yellow) with 100% producer cells, subjected to a 650x daily dilution factor and an osmotic shock between days 4 and 5. **(D)** Time course for the total population density in the same experiments shown in (B). Left: Isolated nodes (2 replicates per condition). Right: Star networks (1 replicate per condition). Red patch: Duration of the osmotic shock.

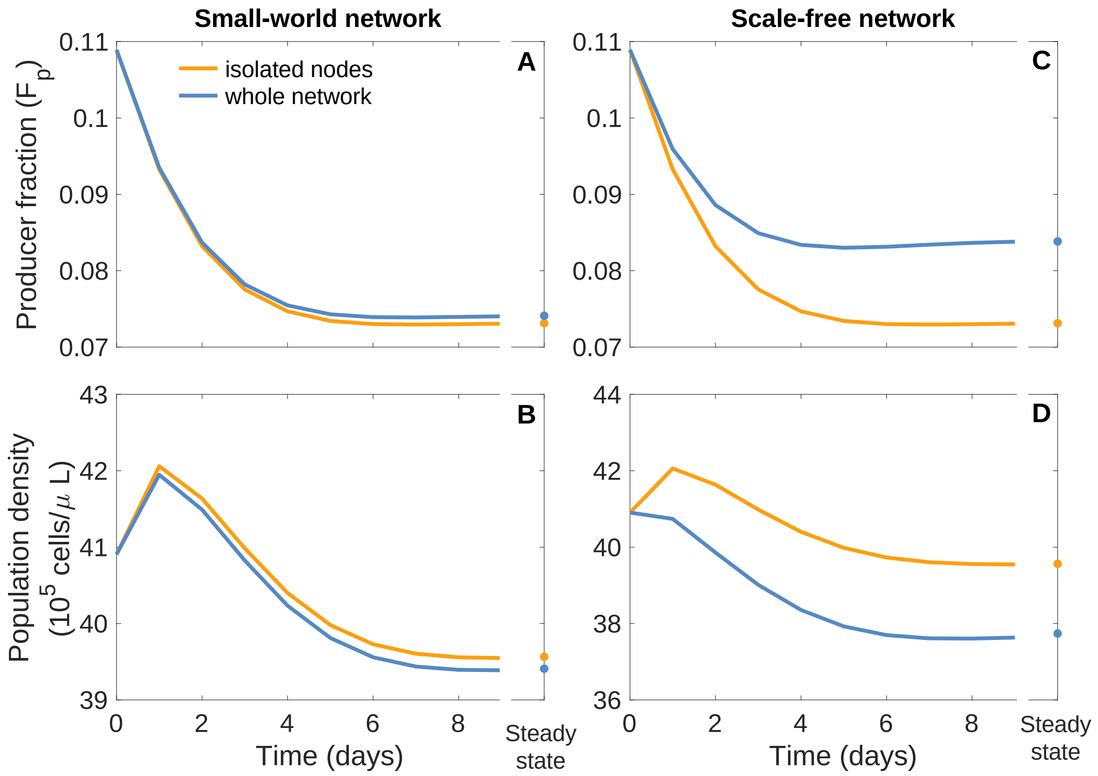

Supplementary Figure 6. Heterogeneous metapopulation structure increases the fraction of producers in complex networks. As the counterparts of the model predications presented in Figures 2D and 2E of the main text, here we show results showing the time evolution of the fraction of producers and density of cells in the small-world (**A**, **B**) and scale-free networks (**C**, **D**). The small-world and scale-free networks are generated by Watts-Strogatz[1] and Barabási-Albert[2] models, respectively, with network size $N = 50$ and average degree $k = 6$. Other model parameters are the same as those given in Fig. 2.

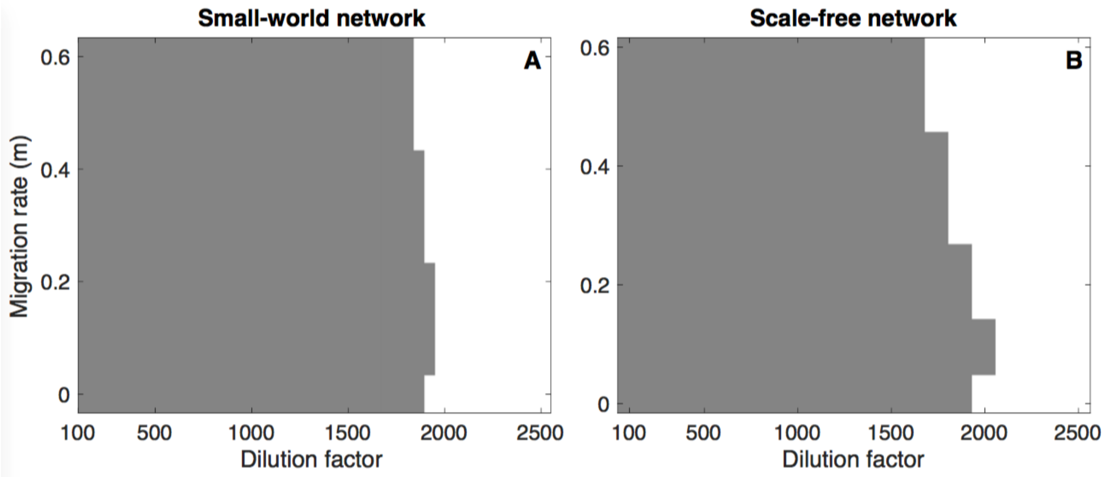

Supplementary Figure 7. Heterogeneous metapopulations represented by small-world and scale-free networks with high migration rates are less able to survive challenging environments than isolated populations. The grey area indicates the conditions (dilution factor and migration rate) in which the model predicts that a 50-node small-world network (**A**) and scale-free network (**B**) will survive after 1000 growth/dilution/migration cycles (note that $m = 0$ is also equivalent to isolated nodes). The rest of model parameters are the same as in Fig. 2. For small migration rate populations with small-world and scale-free networks can survive for larger dilution factors than the isolated populations, but for higher migration rates their survival is worse than isolated populations.

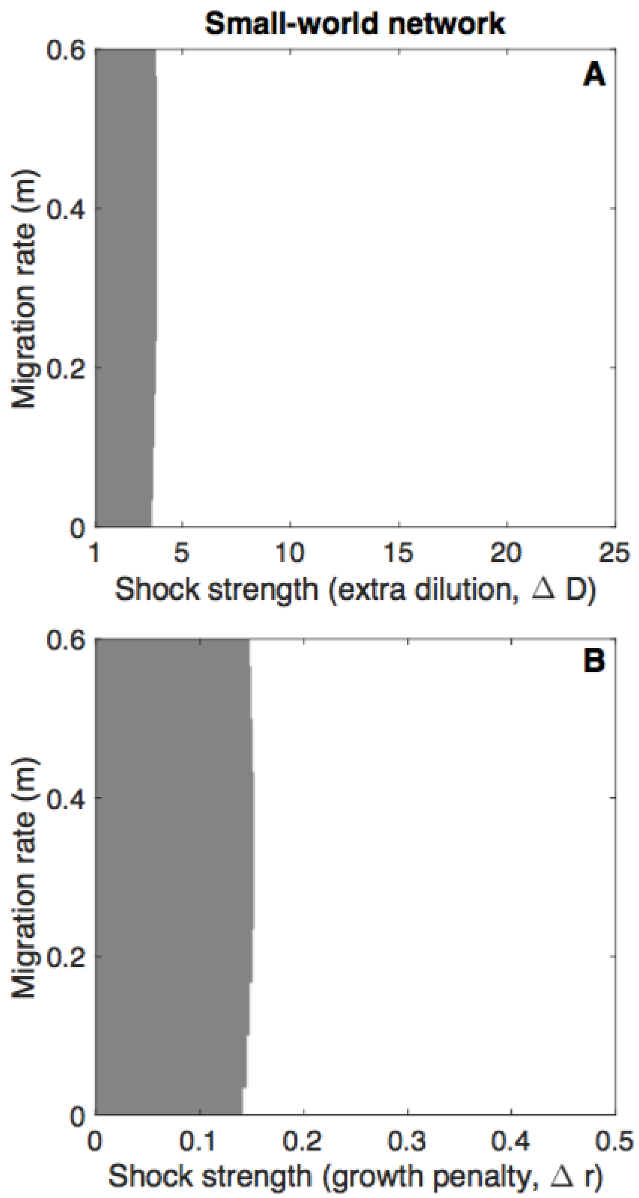

Supplementary Figure 8. Small-world and scale-free networks are more resilient to perturbations than isolated populations. Model prediction for survival of a 50-node small-world (**A**, **B**) and scale-free network (**C**, **D**) with daily dilution factor D=750 after a perturbation, as a function of perturbation strength and migration rate. Rest of parameters are as in Fig. 2. First row: Shock corresponds to one cycle with increased dilution factor, D'=750*ΔD. Second row: Shock corresponds to one cycle with reduced growth r'=0.5-Δr. The increase in resilience is stronger in scale-free networks than in small-world ones. This difference may be due to scale-free networks being more heterogeneous than small-world ones (variance in degree of nodes is 8.9 and 0.84, respectively).

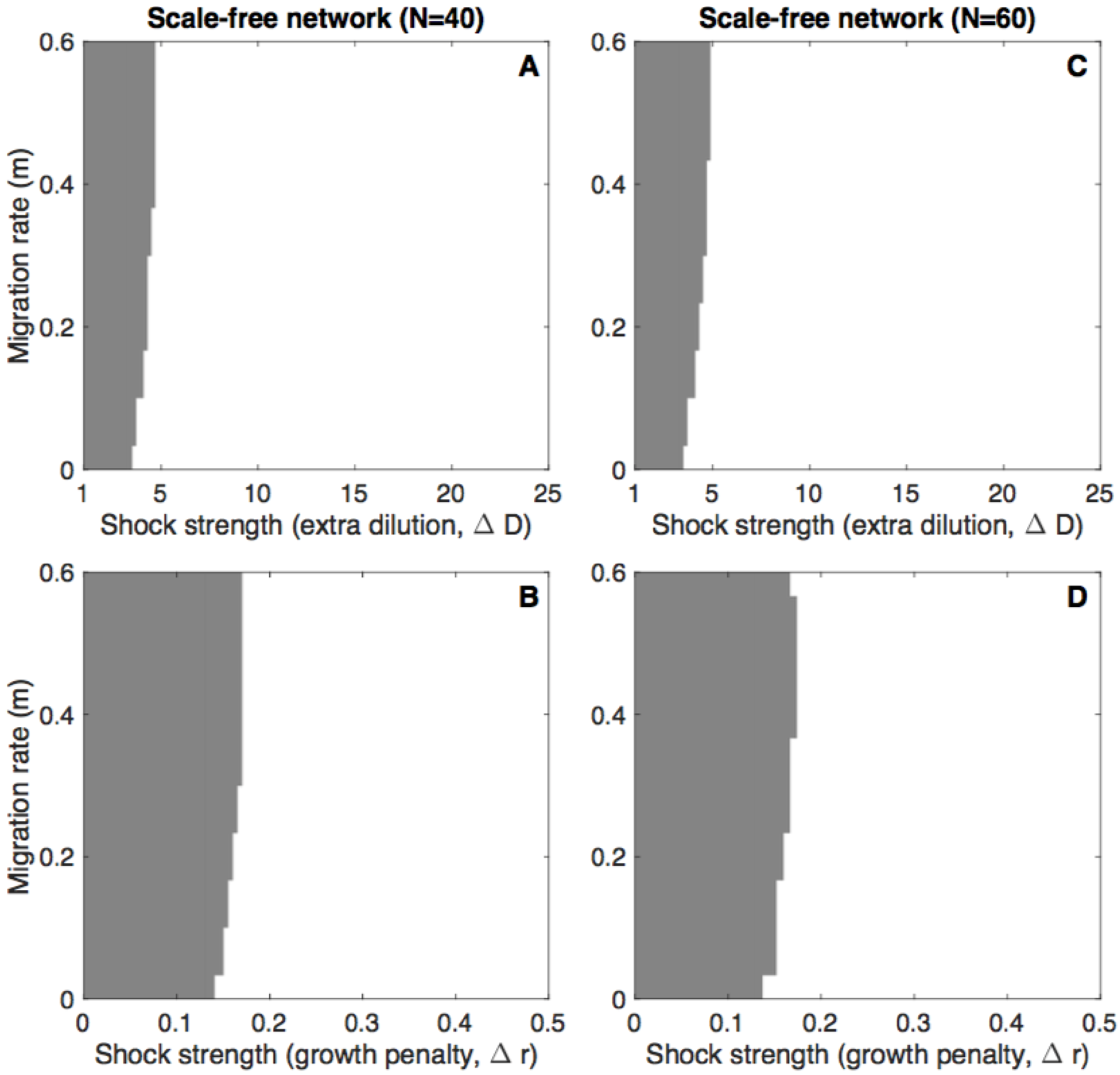

Supplementary Figure 9. Scale-free networks are more resilient to perturbations than isolated populations. Model prediction for survival of a 40-node (**A**, **B**) and 60-node (**C**, **D**) scale-free networks with daily dilution factor D=750 after a perturbation, as a function of perturbation strength and migration rate. Rest of parameters are as those in Fig. S4. First row: Shock corresponds to one cycle with increased dilution factor, D'=750*ΔD. Second row: Shock corresponds to one cycle with reduced growth r'=0.5-Δr.

Supplementary Methods

Dilution-migration protocol

Rationale for the protocol:

In all the experiments in the paper we implement the migration step in parallel with the daily-dilution into fresh media. The protocol can be split into two parts:

1. Fraction $1-m$ = 0.4 cells which stay in the same subpopulation
2. Fraction m = 0.6 cells which migrate outwards from each subpopulation

PART 1:
The 96-well plate after 23 hours of growth (referred to as START) is serially diluted:

1. START plate is diluted 10x in PBS. The new plate is called PBS1 plate.
2. PBS1 plate is diluted again 10x into PBS. The new plate is called PBS2 plate, and is used for flow cytometry
3. PBS1 is diluted in fresh media. The new plate is called RES1 (resident 1, referring to cells which stay in place)
4. RES1 is diluted in fresh media into plate RES2
5. 100 ul from RES2 is taken and added to a fresh 96-well plate (called EXP)

The dilution factors at each step are chosen such that after taking into account the daily dilution factor, 40% of the cells stay in place. (See next section for calculating dilution factors)

PART 2:
Here we deal with the central and side nodes of star network separately:

Central nodes:

1. Here, $m=0.6$ fraction of cells from the center are split equally between all the 9 neighboring side nodes. To do so, small volume of cells in the central node (from PBS1) is diluted into a reservoir containing fresh media. (We used either 10 ml or 25 ml Integra multichannel reservoirs in all experiments, depending on the volume of fresh media)
2. This reservoir (called CEN) is put on a shaker for at least 30 seconds to ensure that the cells are well-mixed.
3. Then using a multichannel pipette, 100 ul is transferred to the 9 side nodes in the EXP plate.

Side nodes:

1. Here, cells are taken from all the side nodes simultaneously (using a multichannel pipette) and added to a reservoir containing fresh media.
2. This reservoir (called SID) is put on a shaker for at least 30 seconds to ensure that the cells are well-mixed.
3. Then using a single-channel pipette, 100 ul is transferred to the central node in the EXP plate.

Fully connected networks:

1. Here, cells are taken from every node in the network (using a multichannel pipette) and added to a reservoir containing fresh media
2. This reservoir (called ALL) is put on a shaker for at least 30 seconds to ensure that the cells are well-mixed.

3. Then using a multichannel pipette, 100 ul is transferred to the entire network in the EXP plate.

Isolated nodes:

1. In part 1, we only accounted for 40% of the cells staying in place. For isolated nodes, all the cells must stay in place, so this step takes care of the other 60% cells.
2. Each row (or column) of isolated nodes is diluted twice in rows (or columns) of a 96-well plate containing fresh media (called the NOTCONN) plate using a multichannel pipette.
3. Using a multichannel pipette, 100 ul is transferred from the second dilution in the NOTCONN plate and transferred to the appropriate row (or column) of the EXP Plate

Calculating the dilution factors and volumes: an example

Here, daily dilution factor is D=650, and *m=0.6*, and N = 10 nodes (i.e. there are 9 side nodes).
Say, we perform a 10 fold dilution into PBS. We add 20 ul of the START Plate to 180 ul of PBS in the PBS1 plate. Similarly, we transfer 20 ul from PBS1 to 180 ul of PBS in the PBS2 plate

PART 1:

- The overall dilution factor in this step is $\frac{D}{20(1-p)} = 81.25$. (The factor of 2 is there because only half of the final volume comes from this part).
- The dilution factor for RES1 and RES2 can be sqrt(81.25), which is ~9. If we transfer 20 ul from each plate to the next, then the volume of media in RES1 and RES2 is 160 ul.

PART 2:

Isolated nodes

- The overall dilution factor in this step is $\frac{D}{20p} = 54.17$
- The dilution factor (for each step) for NOTCONN can be sqrt(54.17), which is 7.35. If we transfer 30 ul during each dilution, then the volume of media in NOTCONN is 191 ul

Central nodes

- The cells are split between 9 side nodes, so the dilution factor is $\frac{D(N-1)}{20p} = 487.5$.
- If we add 10 ul from the central node of PBS1, then the reservoir should have 4875 ul in the reservoir (round off to 4.9 ml)

Side nodes

- Here, we take cells from every side node and pool into a reservoir.
- The dilution factor in this step is $\frac{D}{20p} = 54.17$
- Say we transfer 50 ul from each side node in PBS1 to the reservoir, using a multichannel. For overall dilution factor to be 54.17 the final volume should be ~2.7 ml.
- However, this includes the 50 ul added to the reservoir. For 9 side nodes, we add 450 ul to the reservoir. So the actual volume of fresh media in reservoir is 2.25 mL (round off to 2.3 ml)

Fully connected network

- Here, we take cells from every node in the network and pool into a reservoir.
- The cells are split between 9 neighboring nodes, so the dilution factor is $\frac{D(N-1)}{20p} = 487.5$.
- If we add 10 ul from each node of the network from PBS1 using a multichannel, then the reservoir should have 4775 ul of fresh media in the reservoir (round off to 4.8 ml) to get a final volume of ~4.9ml.

Experiment 1: Increase in cooperator fraction (for daily dilution factor of 650).

Corresponding to Figures 1 and 2

STEPS:

1. Streak the producer (JG300B) and non-producer (JG210C) strains onto YPD Agar plates
2. Pick four colonies of the producer and non-producer each.
3. Grow them overnight in liquid YNB+ Nitrogen, CSM-his, 2% glucose, and 8 μg/mL histidine in a 50-mL Falcon tube at 30C and 50% humidity with shaking at 250 rpm for 24 hours.
4. Mix the two strains at different fractions and dilute them x100 in YNB+Nitrogen + CSM-his supplemented with 2% sucrose, 0.001% glucose and 8 μg/mL histidine.
5. Incubate them for 24 hours at 30C and 50% humidity with shaking at 250 rpm (5 mL of culture in 50 mL Falcon tubes). On the first day of the experiment, determine the fraction of each strain in each co-culture with flow cytometry, and mix different co-cultures in order to achieve the desired starting fraction of producers (f_p) for the experiment.
   Desired starting fractions: 0.1, 0.2, 0.3, 0.4
6. Dilute the co-cultures with the appropriate producer fraction 100 times in YNB+Nitrogen + CSM-his supplemented with 2% sucrose, 0.001% glucose and 8 μg/mL histidine and add to two 96-well plates (one for isolated nodes, another for star and complete networks)
7. Measure OD at 600 nm and incubate for 23 hours at 30C and 50% humidity with 800 rpm shaking.
8. Measure OD the next day at 600 nm.
9. Carry out the transfer protocol as outlined below.
10. Cover the plate with parafilm to limit evaporation.
11. Determine the fraction of producers using flow cytometry after a 100 fold dilution in PBS (make measurements of the PBS2 plate)
12. Measure OD of EXP plate at 600 nm, and incubate the EXP plate at 30C and 50% humidity with 800 rpm shaking.
13. Go to step 8 and repeat till the fraction of producers reaches an equilibrium (should take ~10 days).

NOTE: While doing the transfers from reservoirs or 96 well plates, they are always kept on a shaker, to ensure that we are sampling from a well mixed solution and transfer the correct number of cells.

TRANSFER Protocol:

ISOLATED NODES

Approximate producer fractions f_p :
Row A – 0.1, B – 0.2, C – 0.3, D – 0.4

PREPARATION OF PLATES

- PBS1: Add 180 ul of PBS using 96-well pippetor.
- PBS2: Add 180 ul of PBS using 96-well pippetor to a cold plate.
- RES1: Add 160.0 ul of PBS using 96-well pippetor.
- RES2: Add 160.0 ul of PBS using 96-well pippetor.
- NOTCONN: Add 191.0 ul of PBS using 96-well pippetor.

- CIRCLE: Add 168.0 ul of PBS using 96-well pippetor.

NO RESERVOIRS NEEDED FOR THIS PLATE
RESIDENT TRANSFERS

- Transfer 20 ul from START plate to PBS1 plate.
- Transfer 20 ul from PBS1 plate to PBS2 plate.
- Transfer 20 ul from PBS1 plate to RES1 plate.
- Transfer 20 ul from RES1 plate to RES2 plate.
- Transfer 100 ul from RES2 plate to EXP plate.

MIGRATION
ISOLATED NODES - Rows A-D

- From PBS1, transfer 30 ul from row A of PBS1 to row A of NOTCONN.
- Transfer 30 ul from row A to row B of NOTCONN.
- From PBS1, transfer 30 ul from row B of PBS1 to row C of NOTCONN.
- Transfer 30 ul from row C to row D of NOTCONN.
- From PBS1, transfer 30 ul from row C of PBS1 to row E of NOTCONN.
- Transfer 30 ul from row E to row F of NOTCONN.
- From PBS1, transfer 30 ul from row D of PBS1 to row G of NOTCONN.
- Transfer 30 ul from row G to row H of NOTCONN.
- Transfer 100 ul from row B of NOTCONN to row A of EXP.
- Transfer 100 ul from row D of NOTCONN to row B of EXP.
- Transfer 100 ul from row F of NOTCONN to row C of EXP.
- Transfer 100 ul from row H of NOTCONN to row D of EXP.

10-node STAR and FULLY CONNECTED NETWORKS

Approximate producer fractions f_p :
Row A,E – 0.1, B,F – 0.2, C,G – 0.3, D,H – 0.4

PREPARATION ON PLATES

- PBS1: Add 180 ul of PBS using 96-well pippetor.
- PBS2: Add 180 ul of PBS using 96-well pippetor to a cold plate.
- RES1: Add 160.0 ul of PBS using 96-well pippetor.
- RES2: Add 160.0 ul of PBS using 96-well pippetor.

PREPARATION OF RESERVOIRS

- Add 4900.0 ul of medium to CEN-A
- Add 4900.0 ul of medium to CEN-B
- Add 4900.0 ul of medium to CEN-C
- Add 4900.0 ul of medium to CEN-D
- Add 2300.0 ul of medium to SIDE-A
- Add 2300.0 ul of medium to SIDE-B
- Add 2300.0 ul of medium to SIDE-C
- Add 2300.0 ul of medium to SIDE-D
- Add 4800.0 ul of medium to ALL-E
- Add 4800.0 ul of medium to ALL-F

- Add 4800.0 ul of medium to ALL-G
- Add 4800.0 ul of medium to ALL-H

RESIDENT TRANSFERS

- Transfer 20 ul from START plate to PBS1 plate.
- Transfer 20 ul from PBS1 plate to PBS2 plate.
- Transfer 20 ul from PBS1 plate to RES1 plate.
- Transfer 20 ul from RES1 plate to RES2 plate.
- Transfer 100 ul from RES2 plate to EXP plate.

MIGRATION TRANSFERS
From PBS1 to reservoirs

- Transfer 10 ul from A2 to CEN-A.
- Transfer 10 ul from B2 to CEN-B.
- Transfer 10 ul from C2 to CEN-C.
- Transfer 10 ul from D2 to CEN-D.
- Transfer 50 ul from A3-A11 to SIDE-A using a multichannel.
- Transfer 50 ul from B3-B11 to SIDE-B using a multichannel.
- Transfer 50 ul from C3-C11 to SIDE-C using a multichannel.
- Transfer 50 ul from D3-D11 to SIDE-D using a multichannel.
- Transfer 10 ul from E2-E11 to ALL-E using a multichannel.
- Transfer 10 ul from F2-F11 to ALL-F using a multichannel.
- Transfer 10 ul from G2-G11 to ALL-G using a multichannel.
- Transfer 10 ul from H2-H11 to ALL-H using a multichannel.

From reservoirs to EXP Plate

- Transfer 100 ul from CEN-A to A3-A11 of EXP Plate using a multichannel.
- Transfer 100 ul from CEN-B to B3-B11 of EXP Plate using a multichannel.
- Transfer 100 ul from CEN-C to C3-C11 of EXP Plate using a multichannel.
- Transfer 100 ul from CEN-D to D3-D11 of EXP Plate using a multichannel.
- Transfer 100 ul from SIDE-A to A2 of EXP Plate.
- Transfer 100 ul from SIDE-B to B2 of EXP Plate.
- Transfer 100 ul from SIDE-C to C2 of EXP Plate.
- Transfer 100 ul from SIDE-D to D2 of EXP Plate.
- Transfer 100 ul from ALL-E to E2-E11 of EXP Plate using a multichannel.
- Transfer 100 ul from ALL-F to F3-F11 of EXP Plate using a multichannel.
- Transfer 100 ul from ALL-G to G3-G11 of EXP Plate using a multichannel.
- Transfer 100 ul from ALL-H to H3-H11 of EXP Plate using a multichannel.

Experiment 2: Collapse of populations in challenging environments

Corresponding to Figure 3 and Supplementary Figure 4

STEPS:

1. Streak the producer (JG300B) and non-producer (JG210C) strains onto YPD Agar plates
2. Pick three colonies of the producer and non-producer each.

3. Grow them overnight in liquid YNB+ Nitrogen, CSM-his, 2% glucose, and 8 μg/mL histidine in a 50-mL Falcon tube at 30C and 50% humidity with shaking at 250 rpm for 24 hours.
4. Mix the two strains at different fractions and dilute them x100 in YNB+Nitrogen + CSM-his supplemented with 2% sucrose, 0.001% glucose and 8 μg/mL histidine.
5. Incubate them for 24 hours at 30C and 50% humidity with shaking at 250 rpm (5 mL of culture in 50 mL Falcon tubes). On the first day of the experiment, determine the fraction of each strain in each co-culture with flow cytometry, and mix different co-cultures in order to achieve an approximate starting fraction of $f_p = 0.4$.
6. Dilute the co-cultures with the appropriate producer fraction 100 times in YNB+Nitrogen + CSM-his supplemented with 2% sucrose, 0.001% glucose and 8 μg/mL histidine and add to three 96-well plates (each 96 well plate corresponding to one set of producer and non-producer colony)
7. Measure OD at 600 nm and incubate for 23 hours at 30C and 50% humidity with 800 rpm shaking.
8. Measure OD the next day at 600 nm.
9. Carry out the transfer protocol as outlined below.
10. Cover the plate with parafilm to limit evaporation.
11. Determine the fraction of producers using flow cytometry after a 100 fold dilution in PBS (make measurements of the PBS2 plate) if possible. Measure at the start and end of the experiment at least.
12. Measure OD of EXP plate at 600 nm, and incubate the EXP plate at 30C and 50% humidity with 800 rpm shaking.
13. Go to step 8 and repeat till the fraction of producers reaches an equilibrium (~12-14 days in this experiment).

NOTE: For experiment in Supplementary Figure 4, follow the steps with exception: all cultures are pure cultures of the producer strain (JG300B).

TRANSFER Protocol:

FOR PRODUCER, NON-PRODUCER CO-CULTURES

(For dilution factors 750-2250)
Row A – 750, B – 1000, C – 1150, D – 1300, E – 1450, F – 1600, G – 1750, H – 2250

PREPARATION OF PLATES
- PBS1: Add 190.0 ul of PBS using 96-well pipette.
- PBS2: Add 160.0 ul of PBS using 96-well pipette to a cold plate.

RES1/RES2/NOTCONN:
- To row A, add 117.0 ul of medium.
- To row B, add 138.0 ul of medium.
- To row C, add 150.0 ul of medium.
- To row D, add 160.0 ul of medium.
- To row E, add 170.0 ul of medium.
- To row F, add 180.0 ul of medium.
- To row G, add 189.0 ul of medium.
- To row H, add 217.0 ul of medium.

PREPARATION OF RESERVOIRS

CEN reservoirs

- To reservoir CEN-A, add 2800.0 ul of medium.
- To reservoir CEN-B, add 3700.0 ul of medium.
- To reservoir CEN-C, add 4300.0 ul of medium.
- To reservoir CEN-D, add 4900.0 ul of medium.
- To reservoir CEN-E, add 5400.0 ul of medium.
- To reservoir CEN-F, add 6000.0 ul of medium.
- To reservoir CEN-G, add 6600.0 ul of medium.
- To reservoir CEN-H, add 8400.0 ul of medium.

SIDE reservoirs

- To reservoir SIDE-A, add 1100.0 ul of medium.
- To reservoir SIDE-B, add 1600.0 ul of medium.
- To reservoir SIDE-C, add 1900.0 ul of medium.
- To reservoir SIDE-D, add 2300.0 ul of medium.
- To reservoir SIDE-E, add 2600.0 ul of medium.
- To reservoir SIDE-F, add 2900.0 ul of medium.
- To reservoir SIDE-G, add 3200.0 ul of medium.
- To reservoir SIDE-H, add 4200.0 ul of medium.

RESIDENT DILUTIONS

- Transfer 10 ul from START plate to PBS1 plate.
- Transfer 40 ul from PBS1 plate to PBS2 plate.
- Transfer 20 ul from PBS1 plate to RES1 plate.
- Transfer 20 ul from RES1 plate to RES2 plate.
- Transfer 100 ul from RES2 plate to EXP plate.

(For dilution factors 400 and 650 (rows A and B in respectively))

PREPARATION OF PLATES

- PBS1: Add 180.0 ul of PBS using 96-well pipette.
- PBS2: Add 180.0 ul of PBS using 96-well pipette to a cold plate.

RES1/RES2/NOTCONN:

- To row A, add 121.0 ul of medium.
- To row B, add 160.0 ul of medium.

PREPARATION OF RESERVOIRS

CEN reservoirs

- To reservoir CEN-A, add 3000.0 ul of medium.
- To reservoir CEN-B, add 4900.0 ul of medium.

SIDE reservoirs

- To reservoir SIDE-A, add 1200.0 ul of medium.
- To reservoir SIDE-B, add 2300.0 ul of medium.

RESIDENT DILUTIONS

- Transfer 20 ul from START plate to PBS1 plate.
- Transfer 20 ul from PBS1 plate to PBS2 plate.
- Transfer 20 ul from PBS1 plate to RES1 plate.
- Transfer 20 ul from RES1 plate to RES2 plate.
- Transfer 100 ul from RES2 plate to EXP plate.

(For all dilution factors):

TRANSFERS FROM CENTER

- From central well (column 3) of row (A-H) of PBS1, transfer 10 ul to CEN-(A-H)

TRANSFERS FROM SIDE

- From side wells (column 4-12) of row (A-H) of PBS1, transfer 50 ul using a multichannel pipette to SID-(A-H)

ISOLATED NODES

- From column (1-2), take 25 ul and transfer to NOTCONN plate.
- Mix and transfer 25 ul to an adjacent column using a multichannel pipette.
- Transfer 100 ul from this column to column (1-2) of EXP plate

TRANSFER TO EXPERIMENTAL PLATE

- From CEN-(A-H), transfer 100 ul to the side wells (columns 4-12) of the EXP plate using a multichannel
- From SID-(A-H), transfer 100 ul to the center well (column 3) of the EXP plate using a single channel pipette

FOR PURE PRODUCER POPULATIONS:

Row A – 750, B – 1000, C – 1250, D – 1500, E – 1750, F – 2000, G – 2250, H – 2500

PREPARATION OF PLATES

- PBS1: Add 190.0 ul of PBS using 96-well pipette.
- PBS2: Add 160.0 ul of PBS using 96-well pipette to a cold plate.

RES1/RES2/NOTCONN:

- To row A, add 117.0 ul of medium.
- To row B, add 138.0 ul of medium.
- To row C, add 157.0 ul of medium.
- To row D, add 174.0 ul of medium.
- To row E, add 189.0 ul of medium.
- To row F, add 204.0 ul of medium.
- To row G, add 217.0 ul of medium.
- To row H, add 230.0 ul of medium.

PREPARATION OF RESERVOIRS
CEN reservoirs

- To reservoir CEN-A, add 2800.0 ul of medium.
- To reservoir CEN-B, add 3700.0 ul of medium.
- To reservoir CEN-C, add 4700.0 ul of medium.
- To reservoir CEN-D, add 5600.0 ul of medium.
- To reservoir CEN-E, add 6600.0 ul of medium.
- To reservoir CEN-F, add 7500.0 ul of medium.
- To reservoir CEN-G, add 8400.0 ul of medium.
- To reservoir CEN-H, add 9400.0 ul of medium.

SIDE reservoirs

- To reservoir SIDE-A, add 1100.0 ul of medium.
- To reservoir SIDE-B, add 1600.0 ul of medium.
- To reservoir SIDE-C, add 2200.0 ul of medium.
- To reservoir SIDE-D, add 2700.0 ul of medium.
- To reservoir SIDE-E, add 3200.0 ul of medium.
- To reservoir SIDE-F, add 3700.0 ul of medium.
- To reservoir SIDE-G, add 4200.0 ul of medium.
- To reservoir SIDE-H, add 4800.0 ul of medium.

RESIDENT DILUTIONS

- Transfer 10 ul from START plate to PBS1 plate.
- Transfer 40 ul from PBS1 plate to PBS2 plate.
- Transfer 20 ul from PBS1 plate to RES1 plate.
- Transfer 20 ul from RES1 plate to RES2 plate.
- Transfer 100 ul from RES2 plate to EXP plate.

TRANSFERS FROM CENTER

- From central well (column 3) of row (A-H) of PBS1, transfer 10 ul to CEN-(A-H)

TRANSFERS FROM SIDE

- From side wells (column 4-12) of row (A-H) of PBS1, transfer 50 ul using a multichannel pipette to SID-(A-H)

ISOLATED NODES

- From column (1-2), take 25 ul and transfer to NOTCONN plate.
- Mix and transfer 25 ul to an adjacent column using a multichannel pipette.
- Transfer 100 ul from this column to column (1-2) of EXP plate

TRANSFER TO EXPERIMENTAL PLATE

- From CEN-(A-H), transfer 100 ul to the side wells (columns 4-12) of the EXP plate using a multichannel
- From SID-(A-H), transfer 100 ul to the center well (column 3) of the EXP plate using a single channel pipette

<u>Experiment 3</u>: Resilience of populations – for daily dilution factor of 750

Corresponding to Figure 4 and Supplementary Figure 5

STEPS:

1. Streak the producer (JG300B) and non-producer (JG210C) strains onto YPD Agar plates
2. Pick three colonies of the producer and non-producer each.
3. Grow them overnight in liquid YNB+ Nitrogen, CSM-his, 2% glucose, and 8 μg/mL histidine in a 50-mL Falcon tube at 30C and 50% humidity with shaking at 250 rpm for 24 hours.
4. Mix the two strains at different fractions and dilute them x100 in YNB+Nitrogen + CSM-his supplemented with 2% sucrose, 0.001% glucose and 8 μg/mL histidine.
5. Incubate them for 24 hours at 30C and 50% humidity with shaking at 250 rpm (5 mL of culture in 50 mL Falcon tubes). On the first day of the experiment, determine the fraction of each strain in each co-culture with flow cytometry, and mix different co-cultures in order to achieve an approximate starting fraction of f_p = 0.25.
6. Dilute the co-cultures with the appropriate producer fraction 100 times in YNB+Nitrogen + CSM-his supplemented with 2% sucrose, 0.001% glucose and 8 μg/mL histidine and add to one row of three 96-well plates (each 96 well plate corresponding to one set of producer and non-producer colony)
7. Measure OD at 600 nm and incubate for 23 hours at 30C and 50% humidity with 800 rpm shaking.
8. Measure OD the next day at 600 nm.
9. Carry out the Transfer protocol outlined below (see section – Excluding day of Salt Shock).
10. Cover the plate with parafilm to limit evaporation.
11. Determine the fraction of producers using flow cytometry after a 100 fold dilution in PBS (make measurements of the PBS2 plate).
12. Measure OD of EXP plate at 600 nm, and incubate the EXP plate at 30C and 50% humidity with 800 rpm shaking.
13. Go to step 8 and repeat till the fraction of producers and OD reaches an equilibrium (~5-6 days in this experiment).
14. Then split the single row into 8, and perform the salt shock as outlined (see section - Day of Salt Shock).
15. Cover the plate with parafilm to limit evaporation.
16. Determine the fraction of producers using flow cytometry after a 100 fold dilution in PBS (make measurements of the PBS2 plate).
17. Measure OD of EXP plate at 600 nm, and incubate the EXP plate at 30C and 50% humidity with 800 rpm shaking.
18. Measure OD the next day at 600 nm.
19. Carry out the Transfer protocol outlined below (see section – Excluding day of Salt Shock).
20. Cover the plate with parafilm to limit evaporation.
21. Determine the fraction of producers using flow cytometry after a 100 fold dilution in PBS (make measurements of the PBS2 plate).
22. Measure OD of EXP plate at 600 nm, and incubate the EXP plate at 30C and 50% humidity with 800 rpm shaking.
23. Go to step 18 and track the OD of the populations for at least 8-10 days.

NOTE: For experiment in Supplementary Figure 5, follow the same steps with one exception: all cultures are pure cultures of the producer strain (JG300B).

Day to day transfers (excluding day of salt shock)

NOTE: Before salt shock – do transfers for only one row (for instance row D). This row will be replicated into 8 rows on the day of salt shock.

PREPARATION OF PLATES

- PBS1: Add 180.0 ul of PBS using 96-well pipette.
- PBS2: Add 180.0 ul of PBS using 96-well pipette to a cold plate.
- RES1/RES2/NOTCONN: Add 174 ul of medium using 96-well pipette

PREPARATION OF RESERVOIRS

- CEN reservoirs: To CEN reservoir, add 5600.0 ul of medium.
- SIDE reservoirs: To SIDE reservoir, add 2700.0 ul of medium.

RESIDENT DILUTIONS

- Transfer 20 ul from START plate to PBS1 plate.
- Transfer 20 ul from PBS1 plate to PBS2 plate.
- Transfer 20 ul from PBS1 plate to RES1 plate.
- Transfer 20 ul from RES1 plate to RES2 plate.
- Transfer 100 ul from RES2 plate to EXP plate.

TRANSFERS FROM CENTER

- From central well (column 3) of row (A-H) of PBS1, transfer 10 ul to CEN-A

TRANSFERS FROM SIDE

- From side wells (column 4-12) of row (A-H) of PBS1, transfer 50 ul using a multichannel pipette to SID-A

ISOLATED NODES

- From column (1-2) of PBS1, take 25 ul and transfer to NOTCONN plate.
- Mix and transfer 25 ul to an adjacent column using a multichannel pipette.
- Transfer 100 ul from this column to column 1 of EXP plate

TRANSFER TO EXPERIMENTAL PLATE

- From CEN-(A-H), transfer 100 ul to the side wells of the EXP plate using a multichannel pipette.
- From SIDE-(A-H), transfer 100 ul to the central well of the EXP plate.

Day of salt shock

Prepare separate media for each row supplemented with the following salt concentrations:

For Figure 4:

Row A : Control (no salt)
Row B : 8 g/L
Row C : 16 g/L
Row D : 24 g/L
Row E : 32 g/L
Row F : 40 g/L
Row G : 56 g/L
Row H : 80 g/L

For Supplementary figure 5:

Row A : Control (no salt)
Row B : 24 g/L
Row C : 32 g/L
Row D : 40 g/L
Row E : 48 g/L
Row F : 56 g/L
Row G : 64 g/L
Row H : 80 g/L

Fill up the reservoirs and the RES1/RES2/NOTCONN plates with the salt supplemented media.

PREPARATION OF PLATES (use media supplemented with salt)

- PBS1: Add 190.0 ul of PBS using 96-well pipette.
- PBS2: Add 160.0 ul of PBS using 96-well pipette to a cold plate.
- RES1/RES2/NOTCONN: Add 174 ul of salt supplemented medium using a multichannel pipette

PREPARATION OF RESERVOIRS (use media supplemented with salt)

- CEN reservoirs: To CEN reservoir, add 5600.0 ul of medium.
- SIDE reservoirs: To SIDE reservoir, add 2700.0 ul of medium.

RESIDENT DILUTIONS

- Transfer 20 ul from row D of START plate to every row (A-H) of the PBS1 plate.
- Transfer 20 ul from PBS1 plate to PBS2 plate.
- Transfer 20 ul from PBS1 plate to RES1 plate.
- Transfer 20 ul from RES1 plate to RES2 plate.
- Transfer 100 ul from RES2 plate to EXP plate.

TRANSFERS FROM CENTER

- From central well (column 3) of PBS1, transfer 10 ul to CEN-(A-H)

TRANSFERS FROM SIDE

- From side wells (column 4-12) of PBS1, transfer 50 ul using a multichannel pipette to SID-(A-H)

ISOLATED NODES

- From column (1-2) of PBS1, take 25 ul and transfer to NOTCONN plate.
- Mix and transfer 25 ul to an adjacent column using a multichannel pipette.
- Transfer 100 ul from this column to column (1-2) of EXP plate

TRANSFER TO EXPERIMENTAL PLATE

- From CEN-(A-H), transfer 100 ul to the side wells of the EXP plate using a multichannel pipette.

- From SIDE-(A-H), transfer 100 ul to the central well of the EXP plate.

References

1. Watts, D. J. & Strogatz, S. H. Collective dynamics of ‘small-world’ networks. *Nature* **393,** 440–442 (1998).
2. Barabási, A.-L. & Albert, R. Emergence of Scaling in Random Networks. *Science (80-.).* **286,** 509–512 (1999).
